# High-pressure synthesis of U_2_[CO_3_]_3_ and U[CO_3_]_2_ as potential host phases for uranium in the Earth’s mantle

**DOI:** 10.1038/s42004-026-01911-0

**Published:** 2026-01-30

**Authors:** Dominik Spahr, Lkhamsuren Bayarjargal, Elena Bykova, Maxim Bykov, Gabriel L. Murphy, Philip Kegler, Victor Milman, Nico Giordano, Björn Winkler

**Affiliations:** 1 Goethe University Frankfurt, Institute of Geosciences, Frankfurt, Germany; 2 Goethe University Frankfurt, Institute of Inorganic and Analytical Chemistry, Frankfurt, Germany; 3 Institute of Fusion Energy & Nuclear Waste Management (IFN-2), Forschungszentrum Jülich GmbH, Jülich, Germany; 4 Dassault Systèmes BIOVIA, Cambridge, UK; 5 Deutsches Elektronen-Synchrotron DESY, Hamburg, Germany

**Keywords:** Solid-state chemistry, Geochemistry

## Abstract

It is well established that a significant amount of heat produced in the Earth’s mantle is due to the decay of uranium. However, uranium cannot be incorporated in large amounts into the most common mantle minerals. Here, we suggest that carbonates could be host phases for uranium in carbon-rich mantle lithologies. Two anhydrous uranium carbonates, U_2_[CO_3_]_3_ and U[CO_3_]_2_, were simultaneously synthesized by a reaction of UO_2_ with CO_2_ in a laser-heated diamond anvil cell at 20(1) GPa and 1800(200) K. Their crystal structures were obtained from synchrotron-based single crystal diffraction data and reproduced by density functional theory-based calculations. In U_2_[CO_3_]_3_ trivalent uranium cations are present, while uranium is four-valent in U[CO_3_]_2_. The synthesis of U_2_[CO_3_]_3_ and U[CO_3_]_2_ is a significant extension of the chemistry of uranium compounds and we provide a straightforward synthesis route for a U^III^-containing compound.

## Abstract (web summary)

It is well established that a significant amount of heat produced in the Earth’s mantle is due to the decay of uranium, yet the incorporation of uranium in deep mantle phases remains poorly explored. Here, two chemically simple uranium carbonates (U_2_[CO_3_]_3_ and U[CO_3_]_2_) were synthesized by a reaction of UO_2_ with CO_2_ at lower mantle conditions, revealing that uranium carbonates could be host phases of uranium in carbon-rich lithologies in the Earth’s mantle.

## Introduction

Geoneutrino spectroscopy at the Borexino facility implies that the total radiogenic power produced within the Earth is currently ≈ 20 TW, of which ≈ 8 TW is from the decay of uranium isotopes (^238^U, ^235^U and their daughter isotopes), while the remainder is due to the decay of ^232^Th and ^40^K and their daughter isotopes^1^. Within the experimental uncertainties theses values are in agreement with the lower values obtained from the KamLAND detector^2^. In the Earth’s crust, uranium, as an incompatible element, is mainly found in the oxides uraninite/pitchblende (UO_2+*x*_), the silicate coffinite (USiO_4_), the U-Ti-oxide brannerite (UTi_2_O_6_), and numerous uranyl-containing minerals such as silicates, sulfates or carbonates^3–6^. In the uranyl-containing minerals, the uranium is incorporated as U^VI^ within linear [UO_2_]^2+^-units, but also [UO_2_]^+^-groups with U^V^ are known to exist^3,4,7^. A large subgroup of the uranyl compounds are the uranyl carbonates. Until 2020 up to 40 anhydrous and hydrous naturally occurring minerals hosting [UO_2_]^2+^-groups, having a variety of different crystal structures and hosting different cations, have been found^6^. In addition to these naturally occurring minerals several synthetic uranyl carbonates have been obtained^6^.

For a pyrolytic mantle, it has been suggested that uranium is predominantly incorporated into CaSiO_3_, as no other major silicate or oxide mantle phase can incorporate significant amounts of uranium^8,9^. However, it is well known that the mantle is not homogeneous. The existence of ultra-deep diamonds from the transition zone or the upper part of the lower mantle unambiguously demonstrates that locally other lithologies are present, with carbon concentrations as high as 10,000 ppm^10–12^. In the last years, it has been demonstrated that in addition to the “conventional” *sp*^2^-carbonates with trigonal [CO_3_]^2−^-groups, both *sp*^3^-carbonates, i.e. those with tetrahedrally coordinated carbon, and pyrocarbonates, i.e. those containing [C_2_O_5_]^2−^-groups, may be stable at pressure and temperature conditions of the Earth’s transition zone or lower mantle^13–20^. So, it seems worthwhile to explore if carbon-rich lithologies may provide host phases for uranium and to establish which type of uranium carbonate can be obtained.

The only compound in the system U–C–O that has been structurally characterized until now is rutherfordine ((UO_2_)[CO_3_])^21,22^. The existence of a hydrated uranium(IV)-oxycarbonate has been reported earlier, but its composition and crystal structure remaines unknown^23^. There have been no high-pressure studies of rutherfordine. While the redox state of the mantle is still controversially debated, it is undisputed that with increasing depth the conditions become more reducing ^24,25^. Hence, a viable high pressure host for uranium would need to incorporate the uranium in a reduced form with respect to the U^VI^ in uranyl-groups, and hence rutherfordine can be excluded as a potential uranium host in the deep Earth.

From a crystal chemical perspective, the synthesis of an anhydrous chemically simple uranium carbonate would be of interest for several reasons. Uranium compounds hosting uranium with oxidation states between +2 and +6 have been established^3,26,27^. In addition, the synthesis of a molecular uranium complex with uranium(I) demonstrated quite recently again the chemical variability of uranium in different chemical environments^28^. Chemically simple carbonates have been shown to incorporate cations from alkali metal cations (e.g. Li_2_[CO_3_]) to halogen cations ((IO_2_)_2_[CO_3_])^29,30^, i.e. from +1 to +5, and hence it is not obvious which uranium oxidation state would be preferred. Specifically, the synthesis in a laser-heated diamond anvil cell (LH-DAC) offers the possibility to obtain a carbonate with uranium in a low oxidation state (e.g. +3 or +4) as we have shown in earlier studies, that in reactions of a metal oxide with CO_2_ the valence state of the metal is often preserved. This has been the case during the synthesis of (IO_2_)_2_[CO_3_], Fe_2_[CO_3_]_3_ or Cr_2_[CO_3_]_3_^30–32^.

The synthesis of uranium compounds where uranium is in a low oxidation state has attracted a significant effort in the last few years^26–28,33–36^, and a facile synthesis leading to a new family of uranium compounds would therefore be timely and relevant. Also, it is well established that for anhydrous carbonates with *M*^2+^[CO_3_] composition the ionic radius of the *M*^2+^ cation is a major factor determining if the corresponding carbonate crystallizes in the calcite (*C.N.* = 6) or aragonite (*C.N.* = 9) structure-type^37^. Recently, the first chemically simple anhydrous carbonates, i.e. carbonates with a single type of cation, with trivalent cations have been reported. These include the anhydrous *sp*^2^-carbonates Al_2_[CO_3_]_3_, Fe_2_[CO_3_]_3_, and Cr_2_[CO_3_]_3_, which contain relatively small cations (${r}_{{{\mbox{Fe}}}^{3+}}$ = 0.65 Å, ${r}_{{\mbox{Al}}^{3+}}$ = 0.54 Å ${r}_{{\mbox{Cr}}^{3+}}$ = 0.62 Å in six-fold coordination)^31,32,38,39^. Synthesis of a uranium carbonate containing trivalent uranium (${r}_{{{\mbox{U}}}^{3+}}$ = 1.03 Å)^4,39^ would demonstrate that at high pressures also carbonates with larger trivalent cations can be formed.

As it is an open question whether high-pressure uranium carbonates can be synthesized at *p*,*T*-conditions of the Earth’s mantle and which crystal structure they might have, we investigated the system UO_2_–CO_2_ at moderate pressure. We decided to investigate the reaction at ≈ 20 GPa, as this pressure was applied for the successfully synthesis of the *sp*^2^-carbonates Al_2_[CO_3_]_3_ and Be[CO_3_] from a corresponding oxide (Al_2_O_3_ and BeO) and CO_2_^38,40^. According to the preliminary reference Earth model these pressures would correspond to a depth of ≈ 600 km, inside the lower part of the Earth’s transition zone^41^. The target temperature of the experiment was in the region 1600–2000 K, according to the mantle geotherm at this pressure^42,43^.

## Results and discussion

**Fig. 1:**
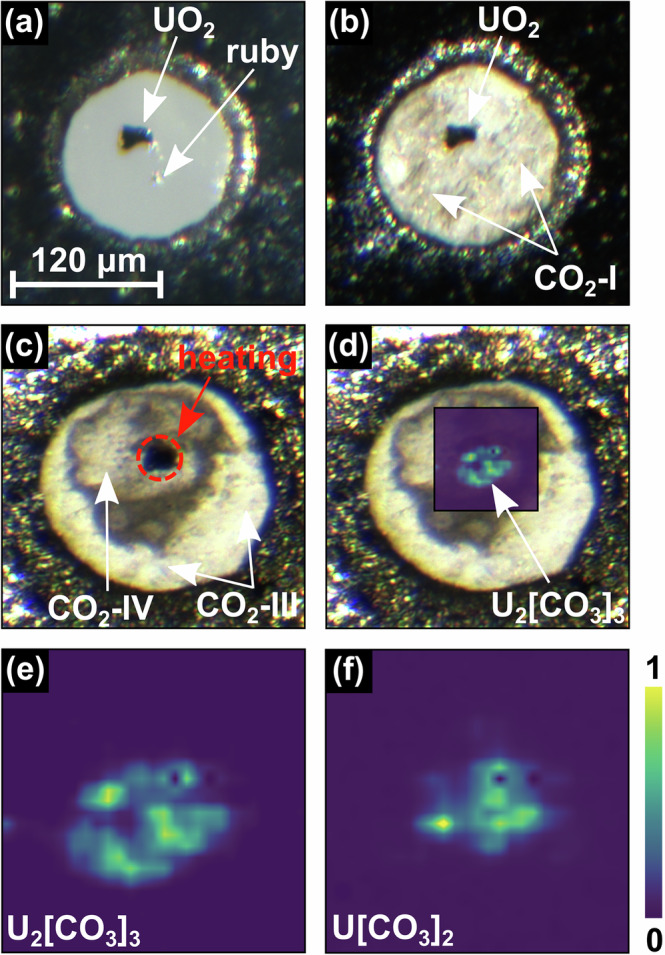
Sample chamber of the DAC and XRD maps of U_2_[CO_3_]_3_ and U[CO_3_]_2_ after the synthesis. **a** UO_2_ single crystal in the sample chamber of the DAC before the cryogenic loading, together with a ruby chip for the pressure determination. **b** CO_2_ was added by cryogenic loading and the DAC was closed tightly. **c** The UO_2_ crystal was laser heated to  ≈ 1800(200) K at 20(1) GPa in the CO_2_ environment. **d** 2D-XRD map of the distribution of U_2_[CO_3_]_3_ as an overlay over the photograph of the sample chamber after the synthesis. XRD-maps of (**e**) U_2_[CO_3_]_3_ (2*θ* ≈ 4.0^∘^) and **f** U[CO_3_]_2_ (2*θ* ≈ 3.7^∘^) measured with *λ* = 0.2900 Å.

The system UO_2_–CO_2_ was investigated using laser heat diamond anvil cells (LH-DACs). The experiments were carried out in analogy to the successful synthesis of the *sp*^2^-carbonates Be[CO_3_], Al_2_[CO_3_]_3_ and Fe_2_[CO_3_]_3_^31,38,40^. First, UO_2_ crystals were selected after their synthesis (see SI) using an optical microscope. In the next step their quality was checked using single crystal X-ray diffraction with a sealed tube X-ray source. Single crystals showing no unexpected reflections were selected for the subsequent experiments. At ambient conditions UO_2_ crystallizes in the cubic space group $Fm\overline{3}m$ with *a* ≈ 5.471 Å depending on the oxygen stoichiometry^44,45^. In a first step, a UO_2_ crystal was placed on the culet of the lower diamond of the DAC (Fig. 1 a). We then added a ruby chip for the determination of the pressure^46^. In a second step, CO_2_-I (dry ice) was cryogenically loaded into the DAC. The DAC was cooled down to ≈ 100 K and CO_2_ was directly condensed into the sample chamber from a gas jet until the sample chamber was filled and the UO_2_ crystal was completely covered. Finally, the DAC was closed tightly (Fig. 1 b). The UO_2_ crystal was compressed to ≈ 20 GPa in the CO_2_ environment, without intermediate heating.

Using Raman spectroscopy we found that after the cryogenic loading CO_2_-I ($Pa\overline{3}$) is present across the sample chamber of the DAC (Fig. 1 b). At low pressures CO_2_-I is the stable polymorph up to its melting temperature, while during pressure increase a phase transition from CO_2_-I to CO_2_-III (*Cmca*) occurs in a broad (≈ 5 GPa) pressure range around ≈ 12 GPa^47–49^. Spatially resolved Raman spectroscopy shows that after increasing the pressure to 20 GPa, but before the laser heating, all CO_2_ is present as CO_2_-III. At the target pressure of the experiment (≈ 20 GPa) the UO_2_ crystal was laser heated in the CO_2_ environment using a CO_2_-laser up to a maximum temperature of 1800(200) K (Fig. 1 c). Heating CO_2_-III at ≈ 20 GPa is expected to cause the appearance of the high-temperature CO_2_-polymorphs CO_2_-II and CO_2_-IV^49–51^.

We employed Raman spectroscopy to detect if a reaction between UO_2_ and CO_2_ had occurred during the laser-heating. We observed that the characteristic Raman modes of the high-temperature CO_2_ polymorphs CO_2_-II and CO_2_-IV (see e.g. Spahr et al. 2024^40^) are present in the sample chamber around the heated area^49–51^. This is consistent with the color change of the CO_2_, visible around the UO_2_ crystal, after the heating (Fig. 1 c). In addition, we observed a weak, but characteristic Raman signal in the region between approximately 1100 cm^−1^ and 1200 cm^−1^. A Raman band at this wavenumber is indicative of the C–O stretching mode in a [CO_3_]^2−^-group of *sp*^2^-carbonates^13,40^.

To gain insights whether novel uranium carbonates were formed during the laser heating, we collected synchrotron X-ray diffraction data on a 2D grid around the heated area in the sample chamber. We found that several reflections are present in the diffraction data which cannot be assigned to known phases. Figure 1 d,e (2*θ *≈ 4.0^∘^) and Fig. 1 f (2*θ* ≈ 3.7^∘^) show XRD-maps of selected unidentified reflections, present in the laser-heated area (measured with *λ* = 0.2900 Å). Due to the large spot size of the CO_2_-laser (30–40 μm) and the long heating time (≈ 30 minutes) the whole UO_2_ crystal and the directly surrounding CO_2_ were heated up to 1800(200) K. As the X-ray maps show that the new phases were exclusively formed in the hottest part, we conclude that their formation requires high temperatures. In a second step, we collected X-ray diffraction data suitable for single crystal structure determination on selected grid points using a ≈ 2 × 2 μm^2^-sized X-ray beam (see SI).

**Fig. 2:**
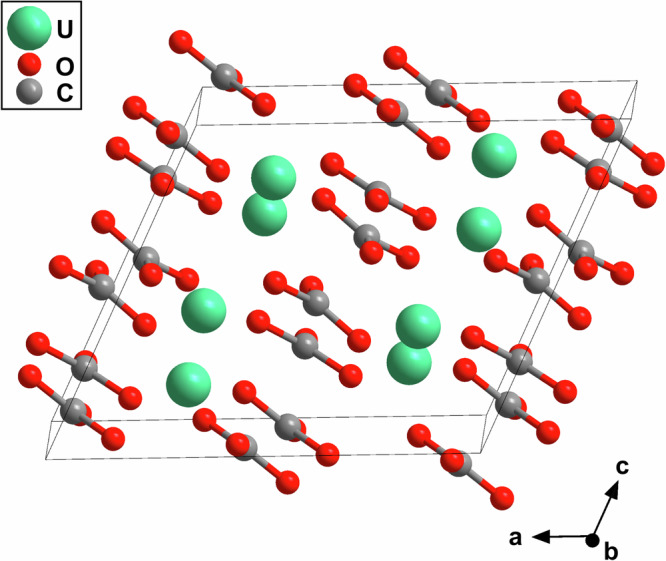
Crystal structure of U_2_[CO_3_]_3_. Monoclinic crystal structure (*C*2/*c*, *Z* = 4) of uranium(III)-carbonate with U_2_[CO_3_]_3_ composition, obtained from synchrotron single-crystal X-ray diffractionat 20(1) GPa.

First, we solved the crystal structure for the phase with strong reflections at 2*θ *≈ 4.0^∘^ (Fig. 2 e). We found that this phase is an uranium(III)-carbonate with U_2_[CO_3_]_3_ composition. At 20(1) GPa it crystallizes in the monoclinic space group *C*2/*c* with *Z* = 4 (Fig. 2). The lattice parameters are *a* = 9.250(5) Å, *b* = 8.0518(7) Å, *c* = 7.837(4) Å and *β* = 115.10(7)^∘^ (*V* = 528.6(5) Å^3^). The very low *R*_1_-value (3.2 %) in combination with a satisfactory reflection-to-parameter-ratio (8.7:1) is indicative of a very good structure refinement. All atomic displacement parameters could be refined anisotropically. This is surprising, as diffraction data are typically dominated by the heavy atoms (*I* ∝ *Z*^2^) and hence the contribution of the carbon and oxygen atoms to the structure factor is small (*Z*_C_ = 6, *Z*_O_ = 8) in comparison to the contribution of the heavy uranium atoms (*Z*_U_ = 92). The peak indexed in the XRD-map at 2*θ *≈ 4.0^∘^ (Fig. 1 d,e) corresponds to the (200) lattice plane. Our DFT-based full geometry optimizations accurately reproduce the experimental structural model (Table S1), confirming our structure solution. We carried out calculations starting with ferromagnetic and anti-ferromagnetic spin configurations. The energetic difference between the optimized structures was only a few meV. Also, the unit cell volumes agreed within the numerical precision. These differences are within the uncertainty of the approach employed here and hence currently we cannot draw any conclusions concerning the magnetic ground state of these compounds.

**Fig. 3:**
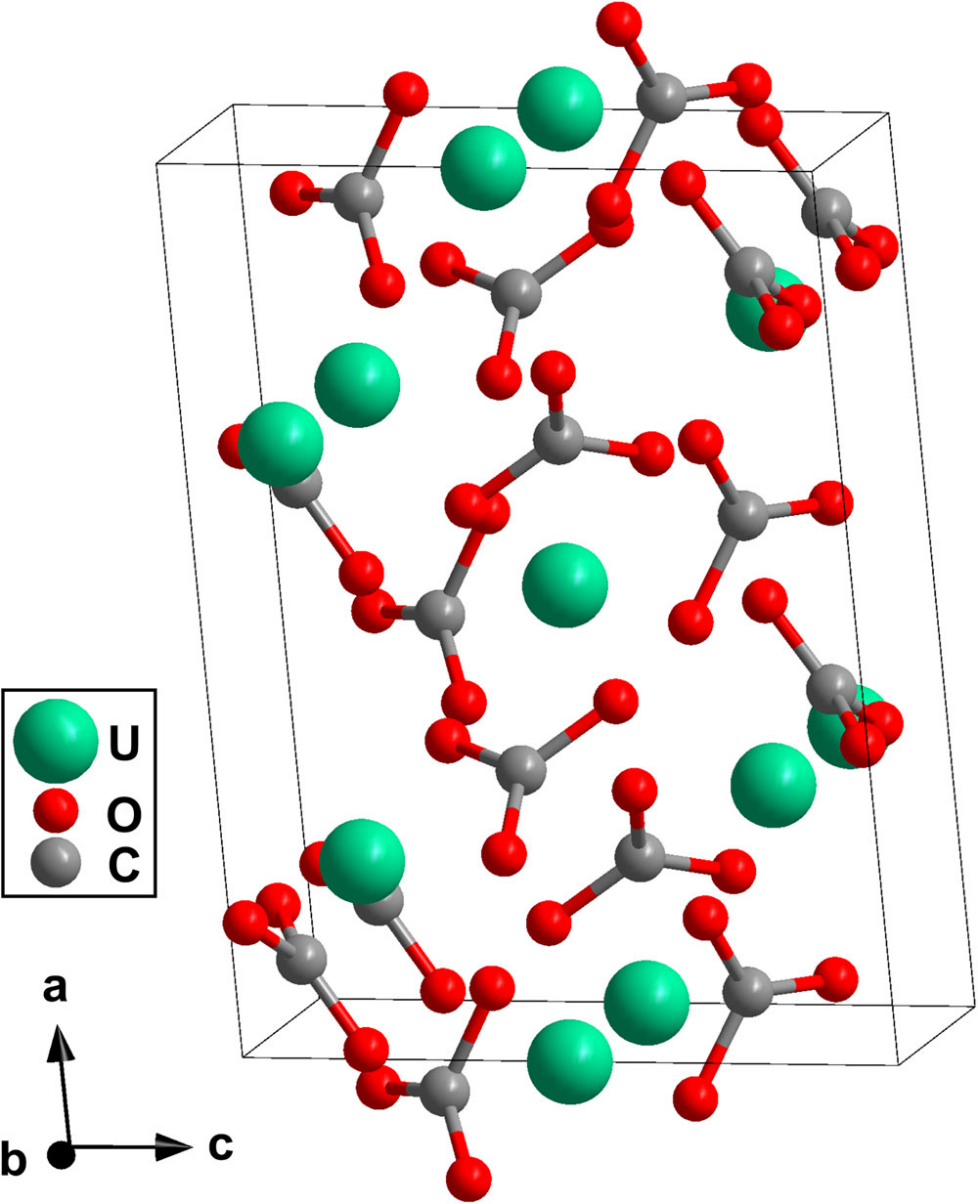
Crystal structure of U[CO_3_]_2_. Monoclinic acentric crystal structure (*C*2, *Z* = 6) of uranium(IV)-carbonate with U[CO_3_]_2_ composition, obtained from synchrotron single-crystal X-ray diffractionat 20(1) GPa.

Afterwards, we analyzed the single crystal diffraction data of the phase with the strong reflection at 2*θ *≈ 3.7^∘^ (Fig. 1 f). From the structure solution we found that this phase is an uranium(IV)-carbonate, U[CO_3_]_2_, which crystallizes in the monoclinic space group *C*2 with *Z* = 6 (Fig. 3). At 20(1) the lattice parameters are *a* = 10.022(2) Å, *b* = 6.597(1) Å, *c* = 7.404(3) Å and *β* = 95.11(2)^∘^ (*V* = 487.6(2) Å^3^). The very low *R*_1_-value (3.8 %) in combination with a very high reflection-to-parameter-ratio for a DAC experiment (17:1) are indicative of a robust structure refinement. However, the atomic displacement parameters of the carbon and oxygen atoms could only be refined isotropically. Similarly to U_2_[CO_3_]_3_, our DFT-based geometry optimizations accurately reproduce the experimental structural model for U[CO_3_]_2_. Specifically, the DFT model retains the acentric space group symmetry and does not converge into a structure with higher space group symmetry (Table S2). This is in agreement with the results using the PLATON/checkCIF program which does not suggest a higher space group symmetry or a centrosymmetric crystal structure. The reflection at 2*θ* ≈ 3.7^∘^ in the XRD-map (Fig. 1 f) corresponds to the ($\overline{1}\overline{1}1$) lattice plane. As in all single-crystal diffraction studies using DACs, the coverage of reciprocal space is limited by the opening angle of the cell. Nevertheless, the reciprocal space reconstruction after data collection demonstrates the high-quality of the collected diffraction data for U_2_[CO_3_]_3_ (Fig. S1) and for U[CO_3_]_2_ (Fig. S2).

Both uranium carbonates synthesized here (U_2_[CO_3_]_3_ and U[CO_3_]_2_) belong to the family of *sp*^2^-carbonates. Their crystal structures are characterized by nearly planar trigonal [CO_3_]^2−^-groups. The arrangement of the [CO_3_]^2−^-groups differs substantially between the two carbonates (Fig. 2 & 3). In U_2_[CO_3_]_3_ the [CO_3_]^2−^-groups are arranged in layers stacked approximately along the *c*-axis. This is in contrast to their arrangement in U[CO_3_]_2_, where several different orientations of the [CO_3_]^2−^-groups can be found. The experimentally observed C–O bond distances within the [CO_3_]^2−^-groups range from 1.24 Å to 1.33 Å (U_2_[CO_3_]_3_) and from 1.24 Å to 1.33 Å (U[CO_3_]_2_), which is in the same range than for other *sp*^2^-carbonates synthesized at similar pressures^20,31,38,40,52^. The C–O bond distances are also in agreement with our DFT-based calculations for U_2_[CO_3_]_3_ (1.27 Å − 1.29 Å) and U[CO_3_]_2_ (1.24 Å − 1.30 Å). Our Mulliken population analysis yielded almost identical values for the C–O bonds in U_2_[CO_3_]_3_ (0.90 e^−^/Å^3^ − 0.97 e^−^/Å^3^) and U[CO_3_]_2_ (0.85 e^−^/Å^3^ − 1.00 e^−^/Å^3^), which are indicative for strong covalent bonds. The Mulliken populations are similar to those obtained for e.g. the C–O bonds in the [CO_3_]^2−^-groups of (IO_2_)_2_[CO_3_] at similar pressures (0.85 e^−^/Å^3^ and 0.87 e^−^/Å^3^)^30^.

**Fig. 4:**
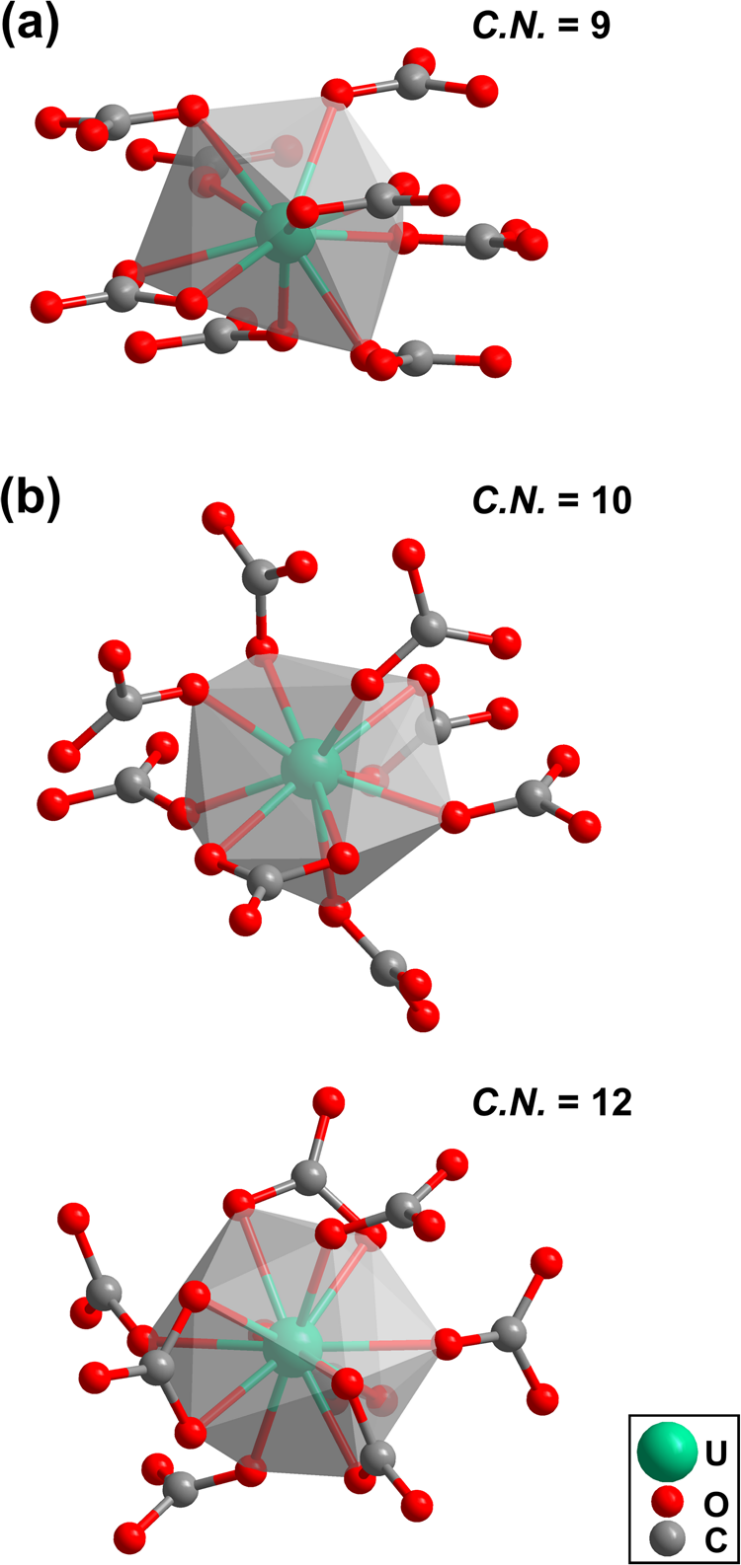
Uranium-oxygen coordination polyhedra in uranium(III)- and uranium(IV)-carbonate. **a** Uranium-oxygen coordination polyhedra of the U^3+^-cations in U_2_[CO_3_]_3_ and **b** of the U^4+^-cations in U[CO_3_]_2_.

The second building block of both uranium carbonates are irregularly shaped uranium-oxygen coordination polyhedra. In U_2_[CO_3_]_3_ the U^3+^-cation is coordinated by 9 oxygen atoms from 8 [CO_3_]^2−^-groups (Fig. 4 a). The effective coordination number is 8.6 and the polyhedral volume is 30.5 Å^3^. In U[CO_3_]_2_ one U^4+^-cation is surrounded by 10 oxygen atoms from 8 [CO_3_]^2−^-groups (Fig. 4 b) with an effective coordination number 9.6 and a polyhedral volume of 29.7 Å^3^. The second U^4+^-cation shows a significantly higher coordination by 12 oxygen atoms, also from 8 [CO_3_]^2−^-groups (Fig. 4 b). The effective coordination number (11.6) and a polyhedral volume (35.2 Å^3^) are noticeably larger than in the other polyhedron. The effective coordination number was obtained using the software package Vesta^53,54^. The range of the U–O bonds is similar in both carbonates, as in U_2_[CO_3_]_3_ the U–O bond distances range from 2.28 Å to 2.59 Å, while in U[CO_3_]_2_ the U–O bond distances range from 2.27 Å to 2.53 Å. Within the experimental uncertainties the average U–O bond distance in U_2_[CO_3_]_3_ and U[CO_3_]_2_ at 20 GPa are very similar to those in the uranium silicate USiO_4_ (≈ 2.22 Å − 2.37 Å) at a similar pressure (≈17 GPa)^55^. Overall, the pressure induced changes in the bond lengths are small, as at ambient conditions U–O bond lengths in USiO_4_ vary from 2.3 Å to 2.4 Å (see discussion in Bauer et al. 2014^56^). In order to benchmark the effective coordination number derived from VESTA, a Mulliken population analysis was carried out. This population analysis shows that in U[CO_3_]_2_ at 20 GPa, we obtain for the U^4+^-cation 12 contacts ranging from 2.30 Å to 2.51 Å, with Mulliken bond populations ranging from 0.27 e^−^/Å^3^ to 0.06 e^−^/Å^3^. For the second symmetrically independent uranium atom, there are 10 contacts, ranging from 2.32 Å to 2.57 Å, but only nine of them have appreciable Mulliken populations ranging from 0.26 e^−^/Å^3^ to 0.06 e^−^/Å^3^. In U_2_[CO_3_]_3_ there is one symmetrically independent U^3+^-cation, which has nine contacts, ranging from 2.41 Å to 2.56 Å. The Mulliken populations range from 0.18 e^−^/Å^3^ to 0.12 e^−^/Å^3^. The Mulliken populations for the U–O contacts are significantly lower than for the covalent C–O bonds in the [CO_3_]^2−^-groups. The same analysis performed on USiO_4_ at ambient pressure revealed 8 U–O bonds (2.34 Å − 2.44 Å), with Mulliken populations from 0.31 e^−^/Å^3^ to 0.13 e^−^/Å^3^.

The DFT calculation clearly show the different charge and spin states of the uranium in the two compounds. In U_2_[CO_3_]_3_, the Mulliken analysis gives a spin of 3*ℏ*/2 and a Mulliken charge of 1.7 *e*, i.e. the uranium is U^3+^. In U[CO_3_]_2_ the spin of the uranium ions is ≈ 2 *ℏ*/2, and the Mulliken charge is 1.98 *e*, consistent with U^4+^ ions. A comparison of the values obtained for the carbonates to those obtained for USiO_4_ supports the identification of U^4+^ in U[CO_3_]_2_ as the spin in uranium in USiO_4_, where it has a formal charge of +4, is ≈ 2.2 *ℏ*/2. The is consistent with the charge balance in the crystal structure for both compounds from the experimental structure solution. Oxygen and carbon atoms carry no spin.

After we established that our DFT-based calculations accurately reproduced our experimentally determined structures for both uranium carbonate phases, we used the calculations to obtain the *p*,*V* relation for U_2_[CO_3_]_3_ and U[CO_3_]_2_ (Fig. S3). In order to determine the bulk modulus *K*_0_ and its pressure derivative *K*_p_ for both phases the calculated *p*,*V* data were fitted separately with an equation of state (EoS). For U_2_[CO_3_]_3_ we obtained a bulk modulus of *K*_0_ = 73(4) GPa with *K*_p_ = 3.6(3). The bulk modulus is similar to that of the second uranium carbonate, U[CO_3_]_2_ (*K*_0_ = 87(3) GPa with *K*_p_ = 5.2(2)), but the pressure dependence *K*_p_ is significantly smaller for U[CO_3_]_2_. The ionic radii of the U^3+^- and U^4+^-cations in the crystal structures of U_2_[CO_3_]_3_ and U[CO_3_]_2_ can be assumed to be in the range between 1.0 Å and 1.2 Å^4,39^. Hence, the bulk moduli of the uranium carbonates synthesized here are in the same range than for other conventional *sp*^2^-carbonates hosting similar sized cations such as Ca[CO_3_]-$R\overline{3}c$ (*r*(Ca^VI^) = 1.00 Å, *K*_0 _≈ 74 GPa), Ca[CO_3_]-*Pmcn* (*r*(Ca^IX^) = 1.18 Å, *K*_0_ ≈ 71 GPa) or Sr[CO_3_]-*Pmcn* (*r*(Sr^IX^) = 1.31 Å, *K*_0_ ≈ 63 GPa)^39,57–60^.

## Conclusion

In summary we synthesized two novel anhydrous uranium carbonates, U_2_[CO_3_]_3_ and U[CO_3_]_2_. The crystal structures of both phases were obtained by synchrotron single crystal X-ray diffraction and confirmed by DFT-based calculations. Both carbonates belong to the family of *sp*^2^-carbonates with [CO_3_]^2−^-groups as the fundamental building unit. We therefore answered the question, whether chemically simple anhydrous *sp*^2^-carbonates with large trivalent or tetravalent cations (*r*(U) ≈ 1.0 Å –1.2 Å)^4,39^ can be formed. This complements earlier studies, where carbonates with small trivalent cations were obtained such as Al_2_[CO_3_]_3_ (*r*(Al^3+^) ≈ 0.5 Å) or Fe_2_[CO_3_]_3_ (*r*(Fe^3+^) ≈ 0.6 Å)^4,31,38,39^. Furthermore, the synthesis route used here is straightforward, in contrast to the experimentally challenging syntheses required for most other U^I^^I^^I^-containing compounds^33,34,36^.

The current experiments were not aimed at obtaining stability fields of the new phases. For this, experiments with large volume presses in conjunction with angle dispersive diffraction are much better suited, as these allow a much superior control of temperature. DFT calculations of uranium-bearing compounds are computationally rather demanding, and currently phonon calculations using spin-polarized approaches with a +U-term are not implemented in our approach. Hence, in the present study, the DFT calculations have only been used to support the analysis of the experimental diffraction data, and to provide a first indication of the compressibility.

The formation conditions of the novel uranium carbonates in the present study (20(1) GPa and 1800(200) K) are compatible with their formation in the Earth’s mantle, especially as the uranium is reduced with respect to its occurrence in uranyl-containing phases^41–43^. The formation of anhydrous uranium bearing carbonates potentially answers the question, what would happen to uranium containing minerals such as uranyl carbonates, which are formed near surfaces by hydrothermal conditions, when they are subducted^4^. In the present study, the discussion focused on uranium with low oxidation states. This is consistent with the assumption that the behavior of U^IV^ dominates that of uranium during mantle processes^61^. However, Gréaux et al. 2012^62^ noted that uranium may exist in high oxidation states metastably in the mantle. It would therefore be also of interest to investigate which phases are formed at mantle conditions when the starting material contains uranyl-groups, but such experiments were outside the scope of the present study. What is required now is to understand the thermodynamic properties of the new carbonates, their stabilities in the presence of mantle minerals such as CaSiO_3_, and their ability to form solid solutions with other high-pressure carbonates, in order to establish into which high pressure phase uranium will preferably partition at high pressures and temperatures.

## Methods

UO_2_ single crystals had been synthesized using a piston cylinder module of a combined piston cylinder/multi anvil apparatus (4 GPa and 1473 K) at the Institute of Nuclear Waste Management (IEK-6) of the Forschungszentrum Juelich from UO_2_ powder, which was prepared using a previously established method^63,64^. The UO_2_ single crystals were loaded in Boehler-Almax type diamond anvil cells (DACs) for the high-pressure experiments^65^ In the next step CO_2_ (Nippon gases, purity ≥99.996%) cryogenically loaded into into the DAC as dry-ice by a custom-built cryogenic loading system^30^. The UO_2_ single crystal was laser-heating from both sides using a custom-built set-up equipped with a Coherent Diamond K-250 pulsed CO_2_ laser (*λ* = 10600 nm) at the target pressure of the experiment^13^. For high pressure Raman spectroscopy, we employed an Oxford Instruments WITec alpha 300R Raman imaging microscope equipped with an Olympus SLMPan N 50 × objective. The measurements were performed using the 532 nm laser and the 1800 grooves mm^−1^ grating of the WITec UHTS 300S (VIS-NIR) spectrograph. High-pressure single-crystal synchrotron X-ray diffraction was carried out at the synchrotron PETRA III (DESY) in Hamburg, Germany, at the extreme conditions beamline P02.2^66^. We used a beam size on the sample of ≈ 2 × 2 μm^2^ (FWHM) and a wavelength of 0.2900 Å (42.7 keV). The diffraction data were collected using a Perkin Elmer XRD1621 detector. First-principles calculations were carried out within the framework of density functional theory (DFT), employing the Perdew-Burke-Ernzerhof (PBE) exchange-correlation functional and the plane wave/pseudopotential approach implemented in the CASTEP simulation package^67–69^. In adition, we employed the correction scheme for van der Waals (v.d.W.) interactions^70^. A detailed description of the experimental and computational methods is available in the supplementary material.

## Supplementary information

Supplemental material for publication

## Data Availability

The X-ray crystallographic coordinates for the structure reported in this study has been deposited at the Cambridge Crystallographic Data Centre (CCDC), under deposition numbers 2475926 (U[CO_3_]_2_) and 2475927 (U_2_[CO_3_]_3_). These data can be obtained free of charge from The Cambridge Crystallographic Data Centre via www.ccdc.cam.ac.uk/data_request/cif. The supplementary material contains additional information to the results of the single crystal structure determination and DFT-based calculations.
