# Supplementary material for: High-pressure synthesis of U2[CO3]3 and U[CO3]2 as potential host phases for uranium in the Earth’s mantle
Source: Commun Chem. 2026 Jan 30;9:112. doi: 10.1038/s42004-026-01911-0 (PMC12960825; doi:10.1038/s42004-026-01911-0)
Supplement: Supplementary file 1 — Supplemental material for publication [file 42004_2026_1911_MOESM1_ESM.pdf]

Supplementary material: High-pressure synthesis of $U_2[CO_3]_3$ and $U[CO_3]_2$ as potential host phases for uranium in the Earth's mantle

Dominik Spahr[*,a], Lkhamsuren Bayarjargal[a], Elena Bykova[a], Maxim Bykov[b], Gabriel Murphy[c], Philip Kegler[c], Victor Milman[d], Nico Giordano[e], Björn Winkler[a]

[a]*Goethe University Frankfurt, Institute of Geosciences, Altenhöferallee 1, 60438 Frankfurt, Germany*
[b]*Goethe University Frankfurt, Institute of Inorganic and Analytical Chemistry, Max-von-Laue-Straße 7, 60438 Frankfurt, Germany*
[c]*Forschungszentrum Jülich · Institute of Energy and Climate Research (IEK), Wilhelm-Johnen-Strasse, 52428 Jülich, Germany*
[d]*Dassault Systèmes BIOVIA, 334 Cambridge Science Park, Cambridge CB4 0WN, United Kingdom*
[e]*Deutsches Elektronen-Synchrotron DESY, Notkestrasse 85, 22607 Hamburg, Germany*

1. Supplementary Methods

1.1. Synthesis of UO_2 single crystals

Uranium dioxide (UO_2) powder, which was prepared using a previously established method,[1] was thoroughly mixed with B_2O_3 using a U:B ratio of 1:10. The mixture was then placed in a sealed platinum crucible and pressurized to $\approx$ 4 GPa followed by heating to 1473 K for 12 hours using a piston cylinder module of a combined piston cylinder/multi anvil apparatus (Voggenreiter LP 1000-540/50) at the Institute of Nuclear Waste Management (IEK-6), Institute of Energy and Climate Research, Forschungszentrum Juelich.[2] The reaction mixture was very slowly cooled to room temperature where upon the pressure slowly released. The platinum crucible was dismantled and fine UO_2 single crystals were mechanically separated and screened using a FEI Quanta 200F scanning electron microscope.

The quality of the UO_2 single crystals was investigated by single-crystal X-ray diffraction using an Oxford Instruments Xcalibur κ-diffractometer. The diffractometer was equipped with a Sapphire 3 CCD detector. The measurements were performed using MoK_α radiation (λ = 0.71073 Å) obtained with a graphite single-crystal monochromator. The beam diameter was $\approx$ 500 µm. The sample-to-detector distance was adjusted to be 42.5 mm. The diffractometer was calibrated by measuring a 152(1) µm ruby sphere (Al_2O_3 with 0.42(1)% Cr_2O_3) from the National Institute of Standards and Technology (NIST SRM 1990).[3]

1.2. High-pressure experiments

The high-pressure experiments were carried out in Boehler-Almax type diamond anvil cells (DACs) equipped on both sides with diamonds having an opening angle of 70° and 350 µm sized culets.[4] We used Re-gaskets, which were pre-indented to thicknesses of $\approx$ 45 µm. Afterwards, sample chambers with $\approx$ 120 µm diameter were drilled into the Re-gaskets using a custom-built laser set-up. The pressure was determined by measuring the shift of the ruby fluorescence and we assume an error of 6% due to non-hydrostatic conditions.[5] We expect that the pressure conditions in the DAC before laser-heating are very likely non-hydrostatic as CO_2-III may sustain pressure gradients up to 0.2 GPa µm^{-1} at high pressures without heating.[6]

In a first step, we added a UO_2 single crystal the sample chamber of the DAC. Afterwards, the DAC was placed on a liquid nitrogen cooled Cu-holder and cooled down for the cryogenic loading using a custom-built cryogenic loading system (see Spahr *et al.*[7]). In a second step, the DAC was cooled down to $\approx$ 100 K and CO_2-I (dry ice) was directly condensed into the gasket hole from the CO_2 (Nippon gases, purity $\geq$ 99.996%) gas jet. We used a small nozzle to direct the CO_2 gas jet with 5 l min^{-1} directly at the gap between the upper diamond and the gasket. Argon as a purge gas to prevent the condensation of H_2O ice. The precipitation of the CO_2 in the gasket hole was monitored using an optical microscope and a camera. After a sufficient amount of CO_2 was gathered in the gasket hole, the DAC was tightly closed and compressed to the target pressure of the experiment.

1.3. Laser heating

The UO_2 single crystal in the CO_2 atmosphere was laser-heated from both sides at the target pressure of the experiment (20(1) GPa) using a custom-built set-up equipped with a Coherent Diamond K-250 pulsed CO_2 laser (λ = 10600 nm).[8] The laser power was adjusted to achieve a coupling of the laser to the sample, using a laser power between 1–6 W. The maximum temperature achieved during the laser-heating was $T_{\rm max} = 1800(200)$ K. The temperatures were determined by the two-color pyrometer method, employing Planck and Wien fits.[9] The heating time during the experiments was $\approx$ 30 min. It is well established that laser-heating in DACs always suffers from large temperature gradients and the actual temperature is strongly dependent on the coupling of the laser with the sample, especially at lower temperatures. At these low temperatures we estimate an uncertainty of at least ±20% of the nominal temperature in the laser-heated region depending on the focus of the laser beam, based on typical 2D temperature-gradient determination experiments performed in DACs.[10]

*d.spahr@kristall.uni-frankfurt.de

1.4. *Raman spectroscopy*

High-pressure Raman spectroscopy was performed in the DACs using an Oxford Instruments WITec alpha 300R Raman imaging microscope. The Raman microscope was equipped with an Olympus SLMPan N 50× objective. The measurements were performed using the 532 nm laser. We employed the 1800 grooves mm^{-1} grating of the WITec UHTS 300S (VIS-NIR) spectrograph in combination with an Andor DR316B-LDC-DD CCD detector for the measurements. The laser power was 100 mW on the sample and the spot size of the Raman laser was ≈ 0.8 μm. We assume a depth resolution of ≈ 6 μm in the direction of the laser beam.

1.5. *Single-crystal synchrotron X-ray diffraction*

Single-crystal synchrotron X-ray diffraction was carried out at the synchrotron PETRA III (DESY) in Hamburg, Germany, at the Extreme Conditions Beamline P02.2.[11] The beam size on the sample was ≈ 2 × 2 μm^2 (FWHM), focused by Kirkpatrick Baez mirrors. The diffraction data were collected using a Perkin Elmer XRD1621 detector, a wavelength of 0.2900 Å (42.7 keV) and a sample to detector distance of 407.3 mm. We rotated the DAC by ±34° around the vertical axis perpendicular to the beam while collecting frames in 0.5° steps with 0.5 s acquisition time per frame. In addition, the intensity of the primary X-ray beam was adjusted by using platinum foil absorbers with different thicknesses (25–150 μm).

The sample to detector distance was calibrated using the powder diffraction pattern of a CeO_2 standard in conjunction with the software DIOPTAS.[12] The diffractometer/detector geometry for the analysis of the single crystal diffraction data was calibrated using diffraction data collected from a single crystal of enstatite ($MgSiO_3$) in a DAC at ambient pressure. After the data collection, the reflections were indexed and integrated employing CrysAlis^{PRO} (version 43.67a).[13] We used the Domain Auto Finder program (DAFi) to find possible single-crystal domains for the subsequent data reduction.[14] The structure solution and refinement were performed using the software package OLEX2 employing SHELXT for the crystal structure determination and SHELXL for the refinement.[15,16,17]

1.6. *Density functional theory-based calculations*

First-principles calculations were carried out within the framework of density functional theory (DFT), employing the Perdew-Burke-Ernzerhof (PBE) exchange-correlation functional and the plane wave/pseudopotential approach implemented in the CASTEP simulation package.[18,19,20] "On the fly" ultrasoft pseudopotentials generated using the descriptors in the CASTEP data base were employed in conjunction with plane waves up to a kinetic energy cutoff of 630 eV. The accuracy of the pseudopotentials is well established.[21] A correction scheme for van der Waals (v.d.W.) interactions was applied in the DFT-calculations. We employed the correction scheme developed by Tkatchenko and Scheffler.[22] A Monkhorst-Pack grid was used for Brillouin zone integrations.[23] We used a distance between grid points of <0.023 $Å^{-1}$. Convergence criteria for geometry optimization included an energy change of $<5 \times 10^{-6}$ eV $atom^{-1}$ between steps, a maximal force of <0.008 eV $Å^{-1}$ and a maximal component of the stress tensor <0.02 GPa. Mulliken charges and bond populations calculations are carried out according to the formalism described by Segall *et al.*[24,25]. The projection of the plane wave states onto a localized basis uses a technique described by Sanchez-Portal *et al.*[26]. The population analysis of the resulting projected states uses the Mulliken formalism.[27]

2. Supplementary Discussion

2.1. Single-crystal synchrotron X-ray diffraction on the uranium(III)-carbonate $U_2[CO_3]_3$

In order to determine the crystal structures of the unknown phases, we used synchrotron X-ray diffraction. We collected X-ray diffraction data on a grid using a spot size of $\approx 2 \times 2$ µm^2 in order to locate promising positions for the collection of single-crystal diffraction data. Afterwards, we collected diffraction data suitable for single-crystal X-ray diffraction analysis on different positions with short exposure times (1 s/°). First, we determined the crystal structure of the unknown phases with a strong reflection at $2\theta \approx 4.7°$. Fig. S 1 a shows the reciprocal space reconstruction for the ($\bar{1}kl$) plane of the crystal on which the crystal structure was determined later. The crystal scatters strongly. In addition to the reflection of the unknown phase, we observed reflections and powder rings of different CO_2 phases (III and V) and of the diamonds in the diffraction data (Fig. S 1 a). Moreover, reflections due to a second unknown phase are present. Fig. S 1 b shows the projection of the reciprocal space along a^* in the Ewald-Explorer in CrysAlis.

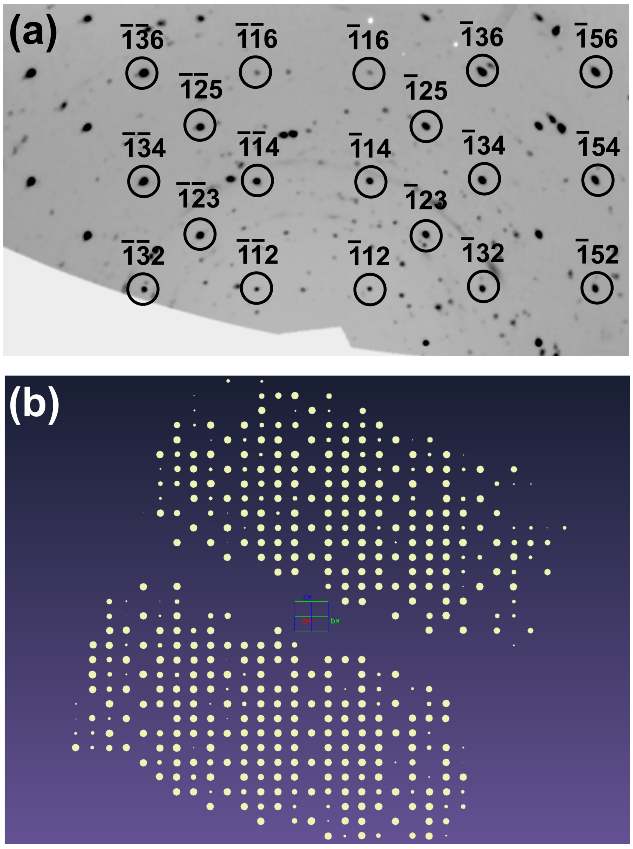

Figure S 1: (a) Reciprocal space reconstruction for the ($\bar{1}kl$) plane of $U_2[CO_3]_3$. (b) Schematic depiction of the reflections in reciprocal space in the Ewald-Explorer in CrysAlis after data reduction which was used for the refinement. The projection of the reciprocal space is shown along a^*.

The crystal structure of the first unknown phase was solved in the centrosymmetric in space group $C2/c$ (No. 15) with $Z = 4$ and $U_2[CO_3]_3$ composition. The very low R_1-value of 3.2% is indicative of a very good structure refinement. In addition, the reflection-to-parameter-ratio (8.7:1) is reasonably high for DAC experiment. By introducing a soft *rigu* constraint for the refinement of the displacement parameters, the displacement parameters of the carbon and oxygen atoms could be refined anisotropically next to the heavy uranium atom. No constraints or restraints for distances or angles had been introduced during the structure refinement. Table 1 lists the experimental crystallographic parameters of $U_2[CO_3]_3$-$C2/c$ at 20(1) GPa in comparison to data derived from the DFT calculations. The unknown reflection at $2\theta \approx 4.0°$ correspond to the (200) lattice plane of $U_2[CO_3]_3$-$C2/c$.

Table S 1: Structural parameters of uranium(III)-carbonate ($U_2[CO_3]_3$) at 20(1) GPa from single-crystal structure solution (ambient temperature) in comparison to data from DFT calculations (athermal limit).

	Single Crystal	DFT
**Crystal data**		
Crystal system	Monoclinic	Monoclinic
Space group	$C2/c$	$C2/c$
Chemical formula	$U_2[CO_3]_3$	$U_2[CO_3]_3$
M_r	656.09	656.09
a (Å)	9.250(5)	9.4099
b (Å)	8.0518(7)	8.1284
c (Å)	7.837(4)	7.7287
α (°)	90.0	90.0
β (°)	115.10(7)	111.54
γ (°)	90.0	90.0
V (Å^3)	528.6(5)	549.84
Z	4	4
**Data collection**		-
F_{000}	1096	-
θ range (°)	2.10–16.80	-
measured reflections	1123	-
independent reflections	566	-
reflections $I > 2\sigma(I)$	483	-
R_{int}	0.018	-
		-
**Refinement**		-
$R_1[I > 2\sigma(I)]$, $wR_2(I)$	0.032, 0.086	-
No. of reflections	137	-
No. of parameters	65	-
No. of restraints	42	-
No. of constraints	0	-
$\Delta\rho_{max}$, $\Delta\rho_{min}$ (e Å^{-3})	2.4, −1.9	-

2.2. *Single-crystal synchrotron X-ray diffraction on the uranium(IV)-carbonate* $U[CO_3]_2$

In order to solve the crystal structures of the second unknown phases we collected X-ray diffraction data at the positions of the X-ray map where mainly unidentified reflections are present. This unknown phase shows a strong reflection at $2\theta \approx 3.7°$. The reciprocal space reconstruction for the ($h0l$) plane of the crystal on which the crystal structure of the second unknown phases was solved is shown in Fig. S 2 a. Again, we observed reflections and powder rings of different CO_2 phases (III and V) and of the diamonds in the diffraction data (Fig. S 2 a). In addition, reflections of $U_2[CO_3]_3$ are present. Fig. S 2 b shows the projection of the reciprocal space along a^* in the Ewald-Explorer in CrysAlis.

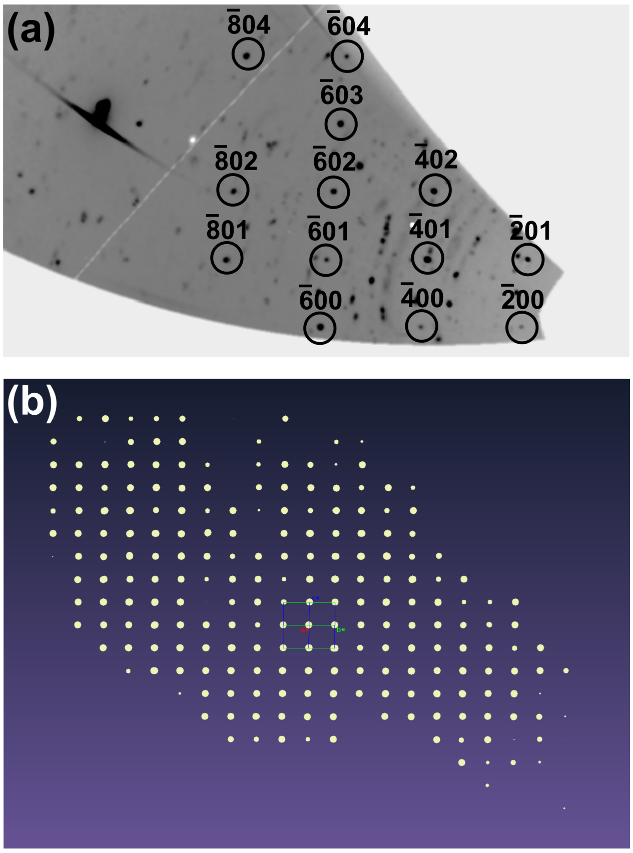

Figure S 2: (a) Reciprocal space reconstruction for the ($h0l$) plane of $U[CO_3]_2$. (b) Schematic depiction of the reflections in reciprocal space in the Ewald-Explorer in CrysAlis after data reduction which was used for the refinement. The projection of the reciprocal space is shown along a^*.

The crystal structure of the second unknown phase was solved in the non-centrosymmetric space group $C2$ (No. 5) with $Z = 6$. The composition was $U[CO_3]_2$. In contrast to $U_2[CO_3]_3$, which contains U^{3+}-cations, $U[CO_3]_2$ hosts U^{4+}-cations. The low R_1-value (3.8%), in combination with a very reflection-to-parameter-ratio (17:1) are indicative of a good structure refinement for DAC experiment. The displacement parameters of the extremely heavy uranium atoms were refined anisotropically, while the displacement parameters of the carbon and oxygen atoms were refined isotropically. No constraints or restraints were applied for the refinement. Table 2 lists the experimental crystallographic parameters of $U[CO_3]_2$-$C2$ at 20(1) GPa in comparison to data derived from the DFT calculations. The unknown reflection at $2\theta \approx 3.7°$ correspond to the ($\bar{1}\bar{1}1$) lattice plane. Testing the structural model of $U[CO_3]_2$-$C2$ using the *PLATON*/checkCIF program does not suggests a higher space group symmetry or a centrosymmetric crystal structure.[28] This is consistent with our DFT-based geometry optimization which retain the acentric space group symmetry.

Table S 2: Structural parameters of uranium(IV)-carbonate ($U[CO_3]_2$) at 20(1) GPa from single-crystal structure solution (ambient temperature) in comparison to data from DFT calculations (athermal limit).

	**Single Crystal**	**DFT**
**Crystal data**		
Crystal system	Monoclinic	Monoclinic
Space group	$C2$	$C2$
Chemical formula	$U[CO_3]_2$	$U[CO_3]_2$
M_r	358.05	358.05
a (Å)	10.022(2)	10.1412
b (Å)	6.597(1)	6.6716
c (Å)	7.404(3)	7.4300
α (°)	90.0	90.0
β (°)	95.11(2)	95.78
γ (°)	90.0	90.0
V (Å^3)	487.6(2)	500.14
Z	6	6
**Data collection**		-
F_{000}	912	-
θ range (°)	1.51–16.83	-
measured reflections	1146	-
independent reflections	1075	-
reflections $I > 2\sigma(I)$	856	-
R_{int}	0.016	-
		-
**Refinement**		-
$R_1[I > 2\sigma(I)]$, $wR_2(I)$	0.038, 0.091	-
No. of reflections	1075	-
No. of parameters	63	-
No. of restraints	1	-
No. of constraints	0	-
$\Delta\rho_{max}$, $\Delta\rho_{min}$ (e Å^{-3})	2.7, −2.2	-

2.3. *Compressibility of $U_2[CO_3]_3$ and $U[CO_3]_2$*

In order to determine the compressibility of $U_2[CO_3]_3$ and $U[CO_3]_2$ we employed our DFT-based calculations in the pressure range between 0 GPa and 30 GPa. The calculated p,V-data were fitted separately with a 3rd-order Birch-Murnaghan equation of states (EoS) in order to determine the bulk modulus (K_0) and its pressure derivative (K_p) using the software package EOSFit7-GUI (Fig. S 3).[29,30,31]

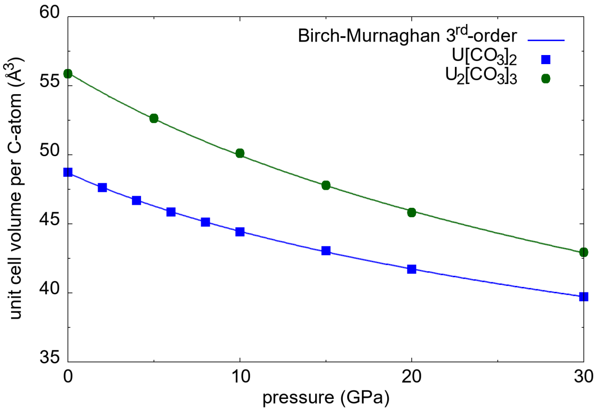

Figure S 3: Fits of Birch-Murnaghan EoS to the p,V-data (0–30 GPa) of $U_2[CO_3]_3$ and $U[CO_3]_2$ obtained by DFT-based calculations. The unit cell volume was normalized with respect to the number of carbon atoms/$[CO_3]^{2-}$-groups in the unit cell.

We derived a theoretical bulk modulus of $K_0 = 73(4)$ GPa with $K_p = 3.6(3)$ for $U_2[CO_3]_3$ in the pressure range between 0–30 GPa from the EoS-fit. The bulk modulus of $U[CO_3]_2$ obtained in the same pressure range range ($K_0 = 87(3)$ GPa with $K_p = 5.2(2)$) is in the same, but the pressure dependency for $U[CO_3]_2$ is significantly higher than for $U_2[CO_3]_3$. From the DFT-based calculations we obtained the densities of both carbonates at 0 GPa. The density of $U[CO_3]_2$ at 0 GPa is 6.1 g cm^{-3}, while that of $U_2[CO_3]_3$ is 6.5 g cm^{-3}. These densities are similar to those of coffinite ($USiO_4$, 6.8 g cm^{-3}) at ambient pressure.[32]

Supplementary References

(1) Murphy, G. L.; Gericke, R.; Gilson, S.; Bazarkina, E. F.; Rossberg, A.; Kaden, P.; Thümmler, R.; Klinkenberg, M.; Henkes, M.; Kegler, P.; Svitlyk, V.; Marquardt, J.; Lender, T.; Hennig, C.; Kvashnina, K. O.; Huittinen, N. Deconvoluting Cr states in Cr-doped UO_2 nuclear fuels via bulk and single crystal spectroscopic studies. *Nat. Commun.* **2023**, *14*, 2455, DOI: 10.1038/s41467-023-38109-0

(2) Murphy, G. L.; Kegler, P.; Alekseev, E. V. Advances and perspectives of actinide chemistry from ex situ high pressure and high temperature chemical studies. *Dalton Trans.* **2022**, *51*, 7401–7415, DOI: 10.1039/D2DT00697A

(3) Wong-Ng, W.; Siegrist, T.; DeTitta, G. T.; Finger, L.; Evans, H. T.; Gabe, E. J.; Enright, G.; Armstrong, J. T.; Levinson, M.; Cook, L. P.; Hubbard, C. Standard Reference MaterialTM (SRM 1990) for Single Crystal Diffractometer Alignment. *J. Res. Natl. Inst. Stand. Technol.* **2001**, *106*, 1071–1094, DOI: 10.6028/jres.106.058

(4) Boehler, R. New diamond cell for single–crystal X-ray diffraction. *Rev. Sci. Instrum.* **2006**, *77*, 115103–1–115103–3, DOI: 10.1029/JB091iB05p04673

(5) Mao, H. K.; Xu, J.; Bell, P. M. Calibration of the ruby pressure gauge to 800 kbar under quasi-hydrostatic conditions. *J. Geophys. Res.* **1986**, *91*, 4673–4676, DOI: 10.1029/JB091iB05p04673

(6) Yoo, C. S.; Cynn, H.; Gygi, F.; Galli, G.; Iota, V.; Nicol, M.; Carlson, S.; Häusermann, D.; Mailhiot, C. Crystal Structure of Carbon Dioxide at High Pressure: "Superhard" Polymeric Carbon Dioxide. *Phys. Rev. Lett.* **1999**, *83*, 5527–5530, DOI: 10.1103/PhysRevLett.83.5527

(7) Spahr, D.; Bayarjargal, L.; Brüning, L.; Kovalev, V.; Bykova, E.; Bykov, M.; Milman, V.; Mezouar, M.; Winkler, B. Synthesis and Crystal Structure of Anhydrous Di-iodyl Carbonate $(IO_2)_2[CO_3]$, Hosting I^{5+}-Cations. *JACS Au* **2025**, *5*, 4675–4680, DOI: 10.1021/jacsau.5c00829

(8) Bayarjargal, L.; Fruhner, C.-J.; Schrodt, N.; Winkler, B. $CaCO_3$ phase diagram studied with Raman spectroscopy at pressures up to 50 GPa and high temperatures and DFT modeling. *Phys. Earth Planet. Inter.* **2018**, *281*, 31–45, DOI: 10.1016/j.pepi.2018.05.002

(9) Benedetti, L. R.; Loubeyre, P. Temperature gradients, wavelength-dependent emissivity, and accuracy of high and very-high temperatures measured in the laser-heated diamond cell. *High Press. Res.* **2004**, *24*, 423–455, DOI: 10.1080/08957950412331331718

(10) Du, Z.; Amulele, G.; Benedetti, L. R.; Lee, K. K. M. Mapping temperatures and temperature gradients during flash heating in a diamond-anvil cell. *Rev. Sci. Instrum.* **2013**, *84*, 075111, DOI: 10.1063/1.4813704

(11) Liermann, H.-P.; Konôpková, Z.; Morgenroth, W.; Glazyrin, K.; Bednarčik, J.; McBride, E. E.; Petitgirard, S.; Delitz, J. T.; Wendt, M.; Bican, Y.; Ehnes, A.; Schwark, I.; Rothkirch, A.; Tischer, M.; Heuer, J.; Schulte-Schrepping, H.; Kracht, T.; Franz, H. The Extreme Conditions Beamline P02.2 and the Extreme Conditions Science Infrastructure at PETRAIII. *J. Synchrotron Radiat.* **2014**, *22*, 908–924, DOI: 10.1107/S1600577515005937

(12) Prescher, C.; Prakapenka, V. B. *DIOPTAS*: a program for reduction of two-dimensional X-ray diffraction data and data exploration. *High. Press. Res.* **2015**, *35*, 223–230, DOI: 10.1080/08957959.2015.1059835

(13) Agilent, CrysAlis PRO, Yarnton, England, **2014**

(14) Aslandukov, A.; Aslandukov, M.; Dubrovinskaia, N.; Dubrovinsky, L. *Domain Auto Finder* (*DAFi*) program: the analysis of single-crystal X-ray diffraction data from polycrystalline sample. *J. Appl. Cryst.* **2022**, *55*, 1383–1391, DOI: 10.1107/S1600576722008081

(15) Dolomanov, O. V.; Bourhis, L. J.; Gildea, R. J.; Howard, J. A. K.; Puschmann, H. *OLEX2*: a complete structure solution, refinement and analysis program. *J. Appl. Cryst.* **2009**, *42*, 339–341, DOI: 10.1107/S0021889808042726

(16) Sheldrick, G. M. *SHELXT* — Integrated space-group and crystal-structure determination. *Acta. Cryst.* **2015**, *A71*, 3–8, DOI: 10.1107/S2053273314026370

(17) Sheldrick, G. M. Crystal structure refinement with *SHELXL*. *Acta. Cryst.* **2015**, *C71*, 3–8, DOI: 10.1107/S2053229614024218

(18) Hohenberg, P.; Kohn, W. Inhomogeneous Electron Gas. *Phys. Rev.* **1967**, *136*, B864–B871, DOI: 10.1103/PhysRev.136.B864

(19) Perdew, J. P.; Burke, K.; Ernzerhof, M. Generalized Gradient Approximation Made Simple. *Phys. Rev. Lett.* **1996**, *77*, 3865–3868, DOI: 10.1103/PhysRevLett.77.3865

(20) Clark, S. J.; Segall, M. D.; Pickard, C. J.; Hasnip, P. J.; Probert, M. I. J.; Refson, K.; Payne, M. C. First principles methods using CASTEP. *Z. Kristallogr.* **2005**, *220*, 567–570, DOI: 10.1524/zkri.220.5.567.65075

(21) Lejaeghere, K.; Bihlmayer, G.; Björkman, T.; Blaha, P.; Blügel, S.; Blum, V.; Caliste, D.; Castelli, I. E.; Clark, S. J.; Dal Corso, A. et al. Reproducibility in density functional theory calculations of solids. *Science* **2016**, *351*, aad3000, DOI: 10.1126/science.aad3000

(22) Tkatchenko, A.; Scheffler, M. Accurate Molecular Van Der Waals Interactions from Ground-State Electron Density and Free-Atom Reference Data. *Phys. Rev. Lett.* **2009**, *102*, 073005, DOI: 10.1103/PhysRevLett.102.073005

(23) Monkhorst, H. J.; Pack, J. D. Special points for Brillouin-zone integrations. *Phys. Rev. B* **1976**, *13*, 5188–5192, DOI: 10.1103/PhysRevB.13.5188

(24) Segall, M. D.; Pickard, C. J.; Shah, R.; Payne, M. C. Population analysis in plane wave electronic structure calculations. *Mol. Phys.* **1996**, *89*, 571–577, DOI: 10.1080/002689796173912

(25) Segall, M. D.; Shah, R.; Pickard, C. J.; Payne, M. C. Population analysis of plane-wave electronic structure calculations of bulk materials. *Phys. Rev. B* **1996**, *54*, 16317–16320, DOI: 10.1103/PhysRevB.54.16317

(26) Sanchez-Portal, D.; Artacho, E.; Soler, J. M. Projection of plane-wave calculations into atomic orbitals. *Solid State Commun.* **1995**, *95*, 685–690, DOI: 10.1016/0038-1098(95)00341-X

(27) Mulliken, R. S. J. Electronic Population Analysis on LCAO–MO Molecular Wave Functions. *J. Chem. Phys.* **1955**, *23*, 1833–1846, DOI: 10.1063/1.1740588

(28) Spek, A. L. Single-crystal structure validation with the program *PLATON*. *J. Appl. Cryst.* **2003**, *36*, 7–13, DOI: 10.1107/S0021889802022112

(29) Murnaghan, F. The Compressibility of Media under Extreme Pressures. *Proc. Natl. Acad. Sci.* **1944**, *30*, 244–247, DOI: 10.1073/pnas.30.9.244

(30) Birch, F. Finite Elastic Strain of Cubic Crystals. *Phys. Rev.* **1947**, *71*, 809–824, DOI: 10.1103/PhysRev.71.809

(31) Gonzalez-Platas, J.; Alvaro, M.; Nestola, F.; Angel, R. *EosFit7-GUI*: a new graphical user interface for equation of state calculations, analyses and teaching. *J. Appl. Cryst.* **2016**, *49*, 1377–1382, DOI: 10.1107/S1600576716008050

(32) Bauer, J. D.; Labs, S.; Weiss, S.; Bayarjargal, L.; Morgenroth, W.; Milman, V.; Perlov, A,; Curtius, H.; Bosbach, D.; Zänker, H.; Winkler, B. High-Pressure Phase Transition of Coffinite, $USiO_4$. *J. Phys. Chem. C* **2014**, *118*, 25141–25149, DOI: 10.1021/jp506368q
